# A Comprehensive Approach to Identify Reliable Reference Gene Candidates to Investigate the Link between Alcoholism and Endocrinology in Sprague-Dawley Rats

**DOI:** 10.1371/journal.pone.0094311

**Published:** 2014-05-13

**Authors:** Faten A. Taki, Abdel A. Abdel-Rahman, Baohong Zhang

**Affiliations:** 1 Department of Biology, East Carolina University, Greenville, North Carolina, United States of America; 2 Department of Pharmacology, East Carolina University, Greenville, North Carolina, United States of America; Cankiri Karatekin University, Turkey

## Abstract

Gender and hormonal differences are often correlated with alcohol dependence and related complications like addiction and breast cancer. Estrogen (E2) is an important sex hormone because it serves as a key protein involved in organism level signaling pathways. Alcoholism has been reported to affect estrogen receptor signaling; however, identifying the players involved in such multi-faceted syndrome is complex and requires an interdisciplinary approach. In many situations, preliminary investigations included a straight forward, yet informative biotechniques such as gene expression analyses using quantitative real time PCR (qRT-PCR). The validity of qRT-PCR-based conclusions is affected by the choice of reliable internal controls. With this in mind, we compiled a list of 15 commonly used housekeeping genes (HKGs) as potential reference gene candidates in rat biological models. A comprehensive comparison among 5 statistical approaches (geNorm, dCt method, NormFinder, BestKeeper, and RefFinder) was performed to identify the minimal number as well the most stable reference genes required for reliable normalization in experimental rat groups that comprised sham operated (SO), ovariectomized rats in the absence (OVX) or presence of E2 (OVXE2). These rat groups were subdivided into subgroups that received alcohol in liquid diet or isocalroic control liquid diet for 12 weeks. Our results showed that U87, 5S rRNA, GAPDH, and U5a were the most reliable gene candidates for reference genes in heart and brain tissue. However, different gene stability ranking was specific for each tissue input combination. The present preliminary findings highlight the variability in reference gene rankings across different experimental conditions and analytic methods and constitute a fundamental step for gene expression assays.

## Introduction

Alcoholism is linked to many different health problems including cardiovascular and neurological impairments and increased cancer risks [1]. It is hard to dissect the mechanism of action of alcohol abuse because it depends on many factors, such as gender, developmental stage, dose, and duration of alcohol consumption [2]. A link between hormones and alcohol dependence was also previously proposed [3]. Studies show that alcohol altered the hormone levels (i.e. progesterone, estrogen) in pre- and post-menopausal females [4] and in ovariectomized monkeys [5].

The significance of endocrinology in the etiology and mechanism of alcohol dependence and addiction has long been discussed [3], [6]. Transient and permanent hormonal changes might be key players in alcohol-associated pathologies such as breast cancer [7]–[10] and neuro-remodeling phenomena like addiction [6]. These alcoholism-related diseases are promoted by fluctuations in gene expressions of some signaling pathways like estrogen and thyroid hormone receptors [9]–[11]. Researchers utilized different model systems, which include mice [12], rats [13], nematodes [14], fruit flies [15] to investigate the pathways involved in mediating alcohol’s impact on the body. Despite the number of studies on ethanol-associated symptoms, many questions remain unanswered and require further investigations on the behavioral, genetic, and biochemical levels.

Quantitative real-time PCR (qRT-PCR) is a gold-standard biotechnique for gene expression analyses. Despite the emergence of the next generation deep sequencing technology, qRT-PCR remains the validation tool of choice. Even though qRT-PCR is a mature biotechnique, it is greatly affected by RNA integrity, purity, and concentration, primer and enzyme efficiencies, genomic DNA contamination, pipetting errors, as well as the choice of proper internal controls (reference genes) [16]. Molecular analyses necessitate reliable normalization to avoid false positive results, which introduce data misinterpretations and imprecise conclusions. An ideal reference gene should have a stable basal expression in different tissues, genders, developmental stages, and experimental conditions and should have similar expression levels to the target genes of interest [17]. So far, there is no one gene whose expression fulfills these criteria [18] although housekeeping genes (HKGs) were widely used as reference genes. The expression levels of HKGs are affected by various experimental conditions [19], [20]. Thus, the identification of suitable reference genes is crucial and should precede gene expression analyses [17]. With this in mind, several statistical approaches have been designed to identify relatively more stable reference genes in response to specific experimental conditions. In this study, we evaluate the stability of 15 commonly used housekeeping genes using 5 statistical methods, which included geNorm [21], delta-Ct (dCt) method [22], NormFinder [23], and BestKeeper [24]. For more accurate ranking of the reference gene candidates, RefFinder was designed to provide a comprehensive ranking [25]. These programs ranked gene candidates based on pairwise comparisons (geNorm, dCt method, BestKeeper) as well as model-based approaches (NormFinder) to determine the most suitable genes.

Alcohol consumption is associated with adverse effects on the cardiovascular and neural systems. Based on the emergent roles of the hormone system in mediating alcohol-induced anomalies, we were interested in understanding the link between alcoholism and estrogen signaling in the heart and brain tissue of Sprague-Dawley rats. For that, we investigated the effect of chronic ethanol treatment on the stability of the expression levels of 15 housekeeping genes in rat heart and brain tissue for identifying most reliable genes as reference genes for gene expression analysis. To perform this study, we treated Sprague-Dawley rats with ethanol (ETOH). The effect of ethanol on the stability of 15 reference gene candidates (Table 1) was investigated using rats with different hormonal backgrounds. The study was based on 6 rat groups. Untreated female (SHAM) and male rats were used as controls. One group of rats underwent ovariectomy (OVX). The last group of rats was ovariectomized and then treated with estrogen hormone (OVXE2). Rats belonging to those two groups were divided into two subgroups. Half of OVX as well as OVXE2 rats served as control (no ETOH treatment), while the remaining rats received ETOH.

**Table 1:** A summary of the 15 HKG (housekeeping genes) considered as reference gene candidates in SD rats.

Genesymbol	Locustag	Genedescription	Forwardprimer (5′→3′)	Reverseprimer (5′→3′)
**18S rRNA**	X01117	18S ribosomal RNA	ACTCAACACGGGAAACCTCA	TCTTAGTTGGTGGAGCGATT
**5S rRNA**	K01594	5S ribosomal RNA	ATCTCGTCTGATCTCGGAA	TCTCCCATCCAAGTACTAACC
**B2m**	NM_012512	beta-2 microglobulin	AGTAGGAGGTGCTCGATGAAG	TCCTGTAGAGCCAGCAACAGG
**BACT**	NM_031144	actin, beta	ACTCTGTGTGGATTGGTGGC	CGCAGCTCAGTAACAGTCCG
**GADD45AF**	NM_024127	growth arrest andDNA-damage-inducible, alpha	TACACTGTGTGCTGGTGACG	ATCACCGTTCGGGGAATCAC
**GAPDH**	NM_017008	glyceraldehyde-3-phosphate dehydrogenase	TGACAACTTTGGCATCGTGG	GGGCCATCCACAGTCTTCTG
**HPRT**	NM_012583	hypoxanthinephosphoribosyltransferase 1	GCCTAAAAGACAGCGGCAAG	GGCTGCCTACAGGCTCATAG
**TBP**	NM_001004198	TATA boxbinding protein	ACCTTATGCTCAGGGCTTGG	GTGCCGTAAGGCATCATTGG
**TNKS**	NM_001106084	TRF1-interactingankyrin-relatedADP-ribose polymerase	CCTACTCCTAGCACATGGCG	AGGTAGGTAAGGCCTCAGGG
**U2**	K00781	small nuclear RNA	ATCTGATACGTCCTCTATCC	GTGGACGGAGCAAGCTCCTA
**U5a**	K00783	small nuclear RNA	ACTCTGGTTTCTCTTCAGATCG	CAGAGTTGTTCCTCTCCA
**U6**	K00784	small nuclear RNA	TTGGAACGATACAGAGAAG	TTTGCGTGTCATCCTTGC
**U87**	AF272707	small nucleolar RNA	ACAATGATGACTTATGTTTTTG	GCTCAGTCTTAAGATTCTC
**UBC**	NM_017314	ubiquitin C	CTCGTACCTTTCTCACCACAGT	GACACCTCCCCATCAAACCC
**Z39**	NR_002705	small nucleolar RNA	GTACATGTGATGAAGCAAATC	TACATCAGAAAGCGTTTACAG

## Materials and Methods

### Animal Handling and Treatment

Animal use and handling protocols were pre-approved and complied with East Carolina University Animal Use and Care Committee guideline. Female and male Sprague-Dawley (SD) rats (9–10 weeks old; Harlan, Indianapolis, UN, USA) were used. Male rats served as control. Female rats were divided into ovariectomized without (OVX) or with and estrogen supplementation (OVXE2) and sham-operated (SO) groups. Ethanol treatment and tissue collection following euthanasia were performed as in our previous studies [26], [27]. Tissue isolation quickly followed. Tissue was flash frozen by liquid nitrogen and then stored at −80°C for subsequent molecular assays.

### Sample Collection and RNA Extractions

Total RNA extraction was performed for heart and brain tissue weighing about 100–200 mg according to protocol using mirVana miRNA Isolation Kit (Life Technologies, CA, USA). Briefly, lysis buffer was added to each sample. The sample was kept on ice while being thoroughly homogenized. Then, an acid-phenol extraction separated RNAs from DNA and proteins. After adding 100% ethanol, the sample-ethanol mixture was passed through a glass-filter by centrifugation. Several washes preceded the elution of the RNA with DNase/RNase-free water. RNA was quantified and its purity was assessed using the NanoDrop ND-1000 Micro-Volume UVVis Spectrophotometer (NanoDrop Technologies, Wilmington, DE).

### Reverse Transcription and qRT-PCR

Reverse transcription was performed using TaqMan microRNA Reverse Transcription kit (Applied Biosystems, Foster City, CA). Poly(T) was used to reverse transcribe the protein coding genes, while specific RT primers were used for the non-coding genes. A total of 1000 ng RNA were used for each RT reaction. RT-PCR was performed in the thermal cycle at 16°C for 30 min followed by 42°C for 30 min, 85°C for 5 min and were finally held at 4°C. For subsequent qRT-PCR, 100 uL DNase/RNase-free water was added to each RT product.

ViiA™ Real-Time PCR System (Applied Biosystem) was used to quantify the expression levels of 15 reference gene candidates on a 384-well-plate. SYBR Green PCR master mix was from SuperArray Bioscience Corp. (Frederick, MD). Specific reverse and forward primers were used (Table 1). Briefly, 5.5 µL DNase/RNase free water, 7.5 µL SYBR Green master mix, 1 µL cDNA (1 ug), 1 µL primer mix were added to each well for a final 15 uL reaction. Four biological replicates were used. Initially, the reaction was set at 95°C for 10 min for enzyme activation and was followed by 40 two-step-cycles of denaturation for 15 sec at 95°C and an annealing/extension step for 60 sec at 60°C.

### Data Analysis

Ct values were exported to an excel file. Descriptive statistics were performed in SPSS (20) and excel. More sophisticated analyses were performed using five statistical approaches: geNorm [21], delta-Ct (dCt) method [22], NormFinder [23], BestKeeper [24] and RefFinder [25].

To prepare geNorm [21] input, the minimal Ct value was used for normalization of all Ct values for each gene across all samples (Ct_original_−Ct_min_). Hence, the lowest value was zero. The difference was then transformed (2^−(Ctoriginal−Ctmin)^). Data was structured such that the gene and sample symbols were in the first row and column, respectively. It was then used as input for geNorm applet. To determine the most stable gene pair, geNorm performs pairwise variation analyses (SD value) for each gene pair across all samples. The software assumes that the genes are not co-regulated and that the transformed expression values of an ideal gene pair are identical across all samples. Then, the geometric mean of the SD values for each gene-related pair combinations is used to compute an M-value. A lower M-value reflects higher gene stability. What follows is a step-wise exclusion of the gene pairs with the highest M-values to reach the most stable gene pair. A beneficial feature of geNorm output is the V-value that reflects the minimal number of genes required for reliable normalization. Such is based on calculating a normalization factor ratio starting with the most stable genes. The program follows a step-wise inclusion process (V_n_/V_n+1_) for more genes until there is no significant change in the normalization factor.

Delta-Ct (dCt) method [22] depends on a concept similar to that of geNorm. However, it does not require a program specialist and can be performed using an excel sheet. This method was designed to overcome limitations associated with small samples like difficulties in using the same standardized mRNA concentrations due to possible protein contaminations. Such technical problems are relieved as genes within one sample are compared to each other by calculating the dCt value. Sensibly, the gene pair with the same dCt value (smallest SD) across all samples is considered to be stable and vice versa. The average of all SD values for each gene set of pairwise combinations is used to rank the gene stability. Genes with lower average SD are more stable than others.

NormFinder [23] is unique as it takes into consideration not only the overall intergroup variation (i.e. control vs. treatment), but also, the intragroup variation (i.e. experimental group replicates). Sample subgroups are taken into account to calculate the most stable gene candidate. Their model adds the two sources of variation to determine the systematic error introduced by the investigated gene. Therefore, this approach is less sensitive to misleading expression patterns for coregulated genes. Meanwhile, the approach takes into account the candidates with less intergroup variations, which might be mistakenly disregarded in the pairwise approaches.

BestKeeper [24] determines the stability of the gene candidates based on the SD of the gene expression levels across samples. Then, genes with least variable Ct values are used for subsequent pairwise comparisons, while those with SD>1 are excluded. The geometric mean of the Ct values of the most highly correlated genes is used to calculate a BestKeeper index. Then, the software calculates Pearson correlation coefficient [r] with a P-value to determine the similarity in the expression levels among the candidates. Thus, genes with least SDs and highest correlation with the index are ranked as the most stable genes. This excel-based applet allows the comparison of only 10 gene candidates. Therefore, we excluded the genes (18S, B2m, BACT, GADD45AF, and TBP) ranked as the least stable using geNorm, dCt method and NormFinder.

RefFinder [25] is another web-based interface that was used to deduce the most stable gene candidates among all methods. For each gene, RefFinder calculates the geometric mean of the ranks calculated by each of the previous approaches. Genes with the lowest rank geometric mean are considered as most stable.

## Results

### Comparing Gene Stabilities by Descriptive Statistics

**Figure 1:**
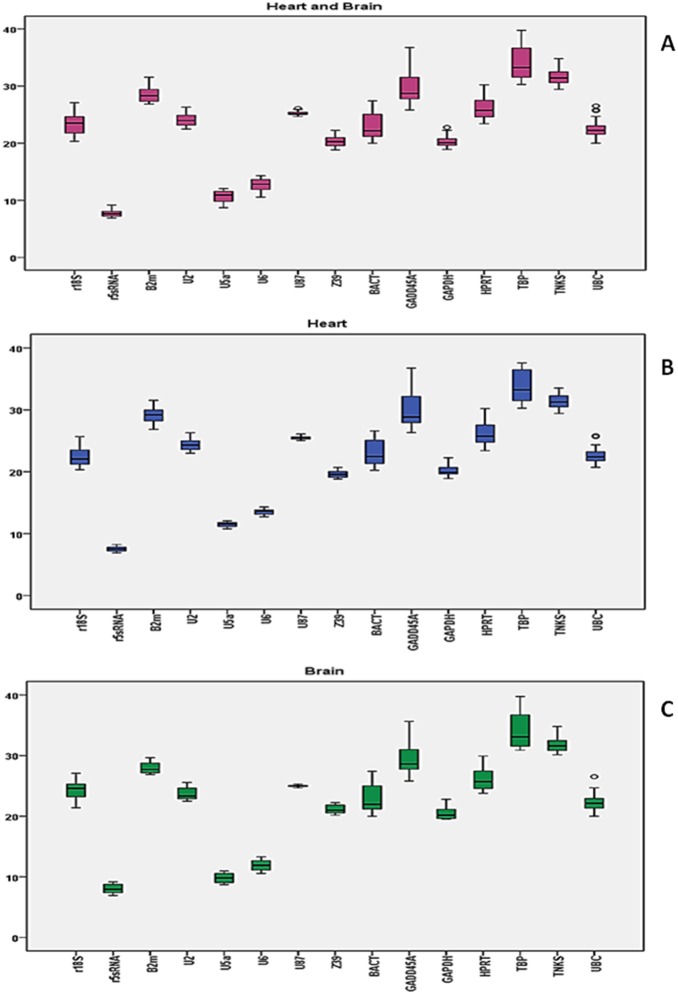
Descriptive statistics of Ct values for heart and brain samples. (A) 48 samples divided into 12 groups for heart and brain tissue combined. (B) 24 samples for 6 groups for heart tissue. (C) 24 samples for 6 groups for brain tissue. Mean Ct values calculated from raw qRT-PCR output for the 15 candidate genes in 6 experimental groups of SD rats (as described in methods). 50% of the values are included in the box. The median is represented by the line in the box. The interquartile range is bordered by the upper and lower edges, which indicate the 75th and 25th percentiles, respectively. The whiskers are inclusive of the maximal and minimal values, but exclusive of the outliers, represented as circles.

We calculated the mean and the standard derivation (SD) of the Ct values for heart and brain samples together, heart samples alone, and brain samples alone. In all combinations, GADD45A, TBP, BACT, 18S rRNA, and HPRT had the most variable expression levels reflected in their high SD values. On the other hand, TBP (Ct_avg_ = 33.86), TNKS (Ct_avg_ = 31.60), GADD45A (Ct_avg_ = 29.69), B2m (Ct_avg_ = 28.53), and HPRT (Ct_avg_ = 26.12) had the highest Ct values and were therefore the least expressed among the gene candidates in the heart and brain (Figure 1; Tables 2 and 3). Thus, TBP, GADD45A, and HPRT are less likely to be good candidates for normalization.

**Table 2:** The mean Ct values for each of the 15 gene candidates in descending order.

H+B	Mean	H	Mean	B	Mean
TBP	33.86	TBP	33.81	TBP	33.90
TNKS	31.60	TNKS	31.35	TNKS	31.84
GADD45A	29.69	GADD45A	29.97	GADD45A	29.42
B2m	28.53	B2m	29.10	B2m	27.95
HPRT	26.12	HPRT	26.16	HPRT	26.07
U87	25.23	U87	25.46	U87	24.99
U2	24.02	U2	24.32	r18S	24.31
r18S	23.39	BACT	23.01	U2	23.72
BACT	22.95	UBC	22.65	BACT	22.89
UBC	22.47	r18S	22.47	UBC	22.29
Z39	20.38	GAPDH	20.17	Z39	21.16
GAPDH	20.32	Z39	19.60	GAPDH	20.47
U6	12.69	U6	13.49	U6	11.90
U5a	10.69	U5a	11.51	U5a	9.87
r5sRNA	7.77	r5sRNA	7.51	r5sRNA	8.03

The values for each input case are shown separately.

**Table 3:** The standard deviations (SD) for each of the 15 gene candidates in descending order.

H+B	SD	H	SD	B	SD
GADD45A	2.67	GADD45A	2.94	TBP	2.73
TBP	2.58	TBP	2.48	GADD45A	2.41
BACT	2.07	BACT	2.05	BACT	2.13
r18S	1.80	HPRT	1.81	HPRT	1.71
HPRT	1.74	r18S	1.51	r18S	1.60
UBC	1.35	UBC	1.30	UBC	1.41
B2m	1.25	B2m	1.28	TNKS	1.28
TNKS	1.22	TNKS	1.14	U2	1.01
U6	1.05	GAPDH	0.90	GAPDH	0.94
U5a	1.02	U2	0.84	B2m	0.93
Z39	1.01	Z39	0.54	U6	0.85
U2	0.97	U6	0.45	U5a	0.76
GAPDH	0.93	r5sRNA	0.41	r5sRNA	0.75
r5sRNA	0.65	U5a	0.37	Z39	0.71
U87	0.32	U87	0.27	U87	0.14

The values for each input case are shown separately.

Regardless of sample combination, the genes with highest expression levels were the same, which included Z39 (Ct_avg_ = 20.38), GAPDH (Ct_avg_ = 20.32), U6 (Ct_avg_ = 12.69), U5a (Ct_avg_ = 10.69), and 5S rRNA (Ct_avg_ = 7.77). However, Z39, U6, 5S rRNA, U5a, and U87 had the least variation in their expression in heart and in brain, respectively. When considering the Ct values from both tissue, Z39, U2, GAPDH, 5S rRNA, and U87 had the lowest SD (Figure 1; Tables 2 and 3). Thus, U87, Z39, and 5S rRNA genes are more stable across groups and in all combinations. With this in mind, we can conclude that Z39 and 5S rRNA are likely to be used for normalization. However, a gene that is more highly abundant than the target genes of interest might mask true changes in expression if used for normalization. On the other hand, we can’t evaluate others like U87 solely based on basic statistics because even though its expression was the least variable, there is still ambiguity in evaluating its relative expression level. Thus, more sophisticated statistical approaches should be employed to evaluate a candidate reference gene.

### Quantitative Analysis of Reference Candidates Based on GeNorm

**Figure 2:**
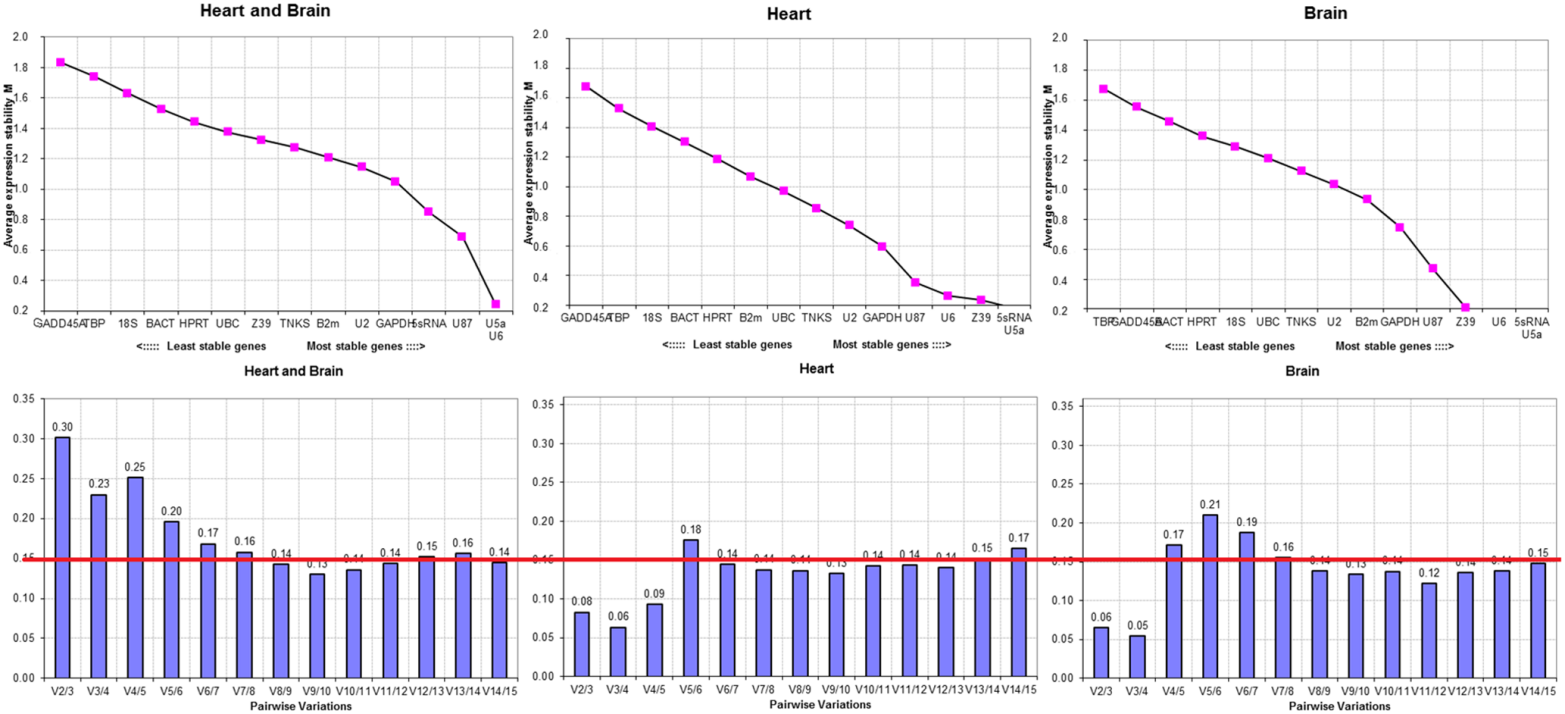
Quantitative and qualitative analysis based on geNorm. (A) Ranking of the 15 gene candidates based on the M-value. Three inputs were used for analysis: Heart and brain combined (48 samples/12 groups), heart alone (24 samples/6 groups), and brain alone (24 samples/6 groups). (B) Determination of the minimal number of reference genes based on V-value for the 3 input combinations. Y-axis represents the ratio of (V_n/_V_n+1_) where 0.15 is the cutoff value. X-axis: V_i/j_ where “i” starts with 2 genes and “j” starts with 3. geNorm starts by a gene pair, and tests whether the inclusion of a 3^rd^ gene adds significant variation. This process is repeated to cover all the genes in the list.

To determine the minimal number of genes required for normalization, we computed the V-value by geNorm. Starting with 2 genes, the software sequentially adds another gene and recalculates the normalization factor ratio. If the added gene does not increase the normalization factor ratio above the cutoff value (0.15), then the original pair of genes is enough for normalization. However, if the new ratio is above 0.15, then more genes should be included. We combined the heart and brain tissue for input in geNorm. The first V-value<0.15 was after V7/8 (Figure 2B). This means that 6 additional genes were required for reliable normalization. The analysis started with a gene pair (i.e. 2 reference genes) and therefore the total would be 8 HKGs for normalization. That accounted for more than 50% of the gene list.

In the following paragraphs, we analyzed the rankings based on 5 statistical methods using the input for combined Ct values from heart and brain tissue. For a higher stringency measure, we only considered the first 6 ranked genes (<50%) for further analyses.

### Determining Best Reference Candidates Based on GeNorm in Both Tissues

GeNorm bases its ranking on the geometric mean of the SD of each transformed gene set of pair combinations (M-value). The lower the M-value is, the higher the ranking. U5a and U6 were co-ranked as most stable genes (M = 0.24). In decreasing order, the third stable gene was U87 (M = 0.69) followed by 5S rRNA (M = 0.85), GAPDH (M = 1.05) and U2 (M = 1.15). The highest M-values ranged between M = 1.32 for UBC and M = 1.83 for GADD45A. Based on M-value, the other genes (HPRT, BACT, 18S rRNA, and TBP) were considered as the least stable genes with M-value between 1.15 to 1.32 (Figure 2A).

### Determining Best Reference Candidates Based on dCt Method in Both Tissues

Gene ranking using the dCt method relies on relative pairwise comparisons. Using raw Ct values, the average SD of each gene set is inversely proportional to gene stability. As shown in Tables 4 and 5, U87 (1.48) was the top-ranked gene. 5s rRNA (1.55) was ranked second and was followed by GAPDH (1.56), UBC (1.59), TNKS (1.64) and U6 (1.69). Oppositely, GADD45A (2.43) 18S rRNA (2.37), and TBP (2.37) were ranked last, while B2m, BACT, and Z39 and were among the less stable genes (1.94–1.78).

**Table 4:** Summary of mean and SD values of gene pairwise comparison using the dCt method for 15 reference gene candidates.

		Pair 1	Pair 2	Pair 3	Pair 4	Pair5	Pair 6	Pair 7	Pair 8	Pair 9	Pair 10	Pair 11	Pair 12	Pair 13	Pair 14	Avg SD
**18S** **rRNA**	Mean	15.6	−5.1	−0.6	12.7	10.7	−1.8	3.0	0.4	−6.3	3.1	−2.7	−10.5	−8.2	0.9	
	SD	1.4	2.6	2.5	2.2	2.1	2.0	1.3	2.9	3.5	2.1	2.8	3.3	2.3	2.3	2.38
**5S** **rRNA**	Mean	−15.6	−20.8	−16.3	−2.9	−4.9	−17.5	−12.6	−15.2	−21.9	−12.6	−18.3	−26.1	−23.8	−14.7	
	SD	1.4	1.8	1.6	1.1	1.1	0.8	0.6	2.1	2.7	1.2	1.8	2.6	1.5	1.4	1.55
**U2**	Mean	0.6	16.3	−4.5	13.3	11.3	−1.2	3.6	1.1	−5.7	3.7	−2.1	−9.8	−7.6	1.5	
	SD	2.5	1.6	0.7	1.5	1.6	0.9	1.8	2.3	2.9	1.1	1.9	2.8	1.5	1.8	1.78
**U5a**	Mean	−12.7	2.9	−17.8	−13.3	−2.0	−14.5	−9.7	−12.3	−19.0	−9.6	−15.4	−23.2	−20.9	−11.8	
	SD	2.2	1.1	1.6	1.5	0.2	0.9	1.6	2.2	2.7	1.6	2.0	2.7	1.8	1.5	1.69
**U6**	Mean	−10.7	4.9	−15.8	−11.3	2.0	−12.5	−7.7	−10.3	−17.0	−7.6	−13.4	−21.2	−18.9	−9.8	
	SD	2.1	1.1	1.6	1.6	0.2	0.9	1.6	2.2	2.7	1.6	2.0	2.7	1.8	1.5	1.69
**U87**	Mean	1.8	17.5	−3.3	1.2	14.5	12.5	4.8	2.3	−4.5	4.9	−0.9	−8.6	−6.4	2.8	
	SD	2.0	0.8	1.1	0.9	0.9	0.9	1.2	2.1	2.7	1.0	1.8	2.6	1.3	1.4	1.48
**Z39**	Mean	−3.0	12.6	−8.1	−3.6	9.7	7.7	−4.8	−2.6	−9.3	0.1	−5.7	−13.5	−11.2	−2.1	
	SD	1.3	0.6	2.1	1.8	1.6	1.6	1.2	2.3	2.9	1.4	2.0	2.7	1.6	1.7	1.77
**B2m**	Mean	5.1	20.8	4.5	17.8	15.8	3.3	8.1	5.6	−1.2	8.2	2.4	−5.3	−3.1	6.1	
	SD	2.6	1.8	0.7	1.6	1.6	1.1	2.1	2.5	3.0	1.4	2.1	3.0	1.7	2.0	1.94
**BACT**	Mean	−0.4	15.2	−5.6	−1.1	12.3	10.3	−2.3	2.6	−6.7	2.6	−3.2	−10.9	−8.6	0.5	
	SD	2.9	2.1	2.5	2.3	2.2	2.2	2.1	2.3	1.4	1.8	1.0	1.0	1.6	1.2	1.90
**GADD45A**	Mean	6.3	21.9	1.2	5.7	19.0	17.0	4.5	9.3	6.7	9.4	3.6	−4.2	−1.9	7.2	
	SD	3.5	2.7	3.0	2.9	2.7	2.7	2.7	2.9	1.4	2.5	1.4	1.9	2.3	1.5	2.44
**GAPDH**	Mean	−3.1	12.6	−8.2	−3.7	9.6	7.6	−4.9	−0.1	−2.6	−9.4	−5.8	−13.5	−11.3	−2.1	
	SD	2.1	1.2	1.4	1.1	1.6	1.6	1.0	1.4	1.8	2.5	1.4	2.3	0.8	1.5	1.55
**HPRT**	Mean	2.7	18.3	−2.4	2.1	15.4	13.4	0.9	5.7	3.2	−3.6	5.8	−7.7	−5.5	3.6	
	SD	2.8	1.8	2.1	1.9	2.0	2.0	1.8	2.0	1.0	1.4	1.4	1.5	1.2	1.1	1.71
**TBP**	Mean	10.5	26.1	5.3	9.8	23.2	21.2	8.6	13.5	10.9	4.2	13.5	7.7	2.3	11.4	
	SD	3.3	2.6	3.0	2.8	2.7	2.7	2.6	2.7	1.0	1.9	2.3	1.5	2.1	1.9	2.36
**TNKS**	Mean	8.2	23.8	3.1	7.6	20.9	18.9	6.4	11.2	8.6	1.9	11.3	5.5	−2.3	9.1	
	SD	2.3	1.5	1.7	1.5	1.8	1.8	1.3	1.6	1.6	2.3	0.8	1.2	2.1	1.5	1.64
**UBC**	Mean	−0.9	14.7	−6.1	−1.5	11.8	9.8	−2.8	2.1	−0.5	−7.2	2.1	−3.6	−11.4	−9.1	
	SD	2.3	1.4	2.0	1.8	1.5	1.5	1.4	1.7	1.2	1.5	1.5	1.1	1.9	1.5	1.59

**Table 5:** A summary of ranking for reference gene candidates using 5 different statistical approaches.

RefFinder		dCt method		NormFinder		geNorm			BestKeeper			
									Ranking			
Genes	GM	Genes	SV	Gene name	SV	Gene name	SV		[r]			SD
**U87**	1.32	U87	1.48	U87	0.76	U5a | U6	0.241	UBC		0.71	U87	0.26
**5sRNA**	2.83	5sRNA	1.55	UBC	0.82	U87	0.688	U6		0.70	5sRNA	0.53
**GAPDH**	3.41	GAPDH	1.56	GAPDH	0.83	5sRNA	0.85	HPRT		0.67	GAPDH	0.74
**U6**	4.14	UBC	1.59	5sRNA	0.89	GAPDH	1.05	U5a		0.65	Z39	0.82
**U5a**	4.28	TNKS	1.64	TNKS	0.94	U2	1.146	5sRNA		0.47	U2	0.84
**UBC**	5.18	U6	1.69	HPRT	1.11	B2m	1.207	U2		−0.35	U5a	0.86
**TNKS**	6.32	U5a	1.7	U6	1.16	TNKS	1.273	TNKS		0.31	U6	0.87
**U2**	7.21	HPRT	1.72	U5a	1.17	Z39	1.323	GAPDH		0.21	TNKS	0.97
**Z39**	7.54	U2	1.77	Z39	1.28	UBC	1.375	U87		0.20	UBC	1.01
**HPRT**	8.73	Z39	1.78	U2	1.28	HPRT	1.441	Z39		0.10	HPRT	1.43
**B2m**	10.02	BACT	1.91	BACT	1.44	BACT	1.526					
**BACT**	11.72	B2m	1.94	B2m	1.53	18S	1.63					
**18S**	13.22	TBP	2.37	TBP	2.06	TBP	1.741					
**TBP**	13.73	18S	2.37	18S	2.06	GADD45A	1.833					
**GADD45A**	14.74	GADD45A	2.43	GADD45A	2.14							

48 samples were combined from heart and brain experimental groups as input.

Geometric mean (GM); Stability Value (SV); Pearson’s correlation coefficient ([r]); Standard Deviation (SD).

### Determining Best Reference Candidates Based on NormFinder in Both Tissues

Complimentary to the pairwise comparisons, NormFinder tests the stability of genes within each sample group as well as between groups. When considering both heart and brain tissue, the total number of sample groups was 12, each having 4 biological replicates. U87 (0.76) was the gene with the least inter and intra-variation in expression levels; thus, U87 would be the most reliable reference gene. The stability values ranged from 0.82 to 1.11 for the other 5 candidate genes (UBC, GAPDH, 5S rRNA, TNKS, and HPRT) (Table 5). Interestingly, based on geNorm, UBC and HPRT were among the least stable genes. This result is based on low intragroup, yet similar intergroup variation. Recalling the model-based approach, NormFinder prevents the exclusion of genes which might have consistent intergroup expression levels. Not necessarily ‘similar’, these genes have ‘minimal’ intergroup as well as intragroup variation. Nevertheless, a drawback in NormFinder is the requirement of a minimum of 8 samples/group. For many gene expression studies including our own, it is challenging to have such a large sample size. Taken together, the differences in methodologies might be a reason behind the inclusion of these two genes among the most stable candidates.

### Determining Best Reference Candidates Based on BestKeeper in Both Tissues

Due to the input size limitation, BestKeeper only analyzed 10 genes, which were ranked the most stable based on other three programs (geNorm, dCt method, and Normfinder). BestKeeper provided a two-way ranking, which separated the correlation of expression among the genes from the overall variations in expression levels (SD). From each approach, we considered the top 3 genes. Those computed to be highly correlated with p-values <0.05 were UBC ([r] = 0.71), U6 ([r] = 0.70), and HRPT ([r] = 0.67) at p = 0.001. On the other hand, based on BestKeeper, U87 (SD = 0.26), 5S rRNA (SD = 0.53), and GAPDH (SD = 0.74) had the least variable expression levels across all heart and brain samples. 5S rRNA was fairly but significantly correlated with the other genes ([r] = 0.47, p = 0.001), while the weaker correlation of U87 and GAPDH was not statistically significant (Table 6). Statistically speaking, this trend is sensible. When the homogeneity of a group increases, the variance (SD) decreases as in the case of U87, 5S rRNA, GAPDH, and Z39 and [r] tends to zero [28]. In fact, BestKeeper calculated the least SD values for these 4 genes. Thus, these genes will share less variation with the others in pairwise variation and will therefore have the least correlation coefficient. That does not render them unsuitable as reference genes candidates and stresses on the importance of taking both criteria ([r] and SD) to choose the best candidate(s). The inclusion of the top three from the [r]-based and SD-based ranking was consistent with 4 out of 6 best ranked genes in geNorm, and 5 out of 6 top genes in dCt-method and NormFinder (Table 5). In addition, consistent with NormFinder, UBC and HPRT were also ranked among the top 6 by BestKeeper (Table 6). Comparing among the different methodologies helped remove the doubt in NormFinder’s result which might have aroused from its requirement of a larger sample size.

**Table 6:** Ranking of 10 reference gene candidates based on BestKeeper.

	n	GM [Ct]	AM [Ct]	min [Ct]	max [Ct]	SD [± Ct]	CV [% Ct]	[r]	p-value	Ranking	
										[r]	SD
**5sRNA**	48	7.74	7.77	6.91	9.20	0.53	6.77	0.47	0.001	UBC	U87
**U2**	48	24.00	24.02	22.48	26.30	0.84	3.48	−0.35	0.015	U6	5sRNA
**U5a**	48	10.64	10.69	8.71	12.06	0.86	8.05	0.65	0.001	HPRT	GAPDH
**U6**	48	12.65	12.69	10.57	14.30	0.87	6.85	0.70	0.001	U5a	Z39
**U87**	48	25.22	25.23	24.68	26.10	0.26	1.03	0.20	0.177	5sRNA	U2
**Z39**	48	20.36	20.38	18.83	22.26	0.82	4.02	0.10	0.487	U2	U5a
**GAPDH**	48	20.30	20.32	18.90	22.78	0.74	3.66	0.21	0.151	TNKS	U6
**HPRT**	48	26.06	26.12	23.40	30.22	1.43	5.46	0.67	0.001	GAPDH	TNKS
**TNKS**	48	31.57	31.60	29.43	34.77	0.97	3.07	0.31	0.034	U87	UBC
**UBC**	48	22.43	22.47	19.99	26.52	1.01	4.48	0.71	0.001	Z39	HPRT

Two criteria are considered: Pearson’s correlation coefficient and BestKeeper computed SD values. The stability of a gene is directly proportional to the [r] value, while it is inversely proportional to the SD value.

Note: Total sample number (n), Geometric Mean (GM), AM (Arithmetic Mean), Standard Deviation (SD), Coefficient of Varience % (CV), Pearson’s correlation coefficient [r], P<0.05.

### Comprehensive Ranking of Best Reference Genes Using RefFinder in Both Tissues

Based on the geometric mean (GM) of the rankings obtained from 4 complementary statistical approaches, U87 was the preferred candidate (GM = 1.32). The remaining highly ranked candidates were 5S rRNA, GAPDH, U6, U5a, and UBC with GM values ranging from 2.83 to 5.18, respectively. On the other hand, B2m, BACT, 18S rRNA, TBP, and GADD45A all had GM values higher than 10 (Table 5). These 5 candidates had the lowest ranking and less likely to serve as reliable reference genes for normalizing gene expression.

### RefFinder Ranking of Gene Candidates for Heart Hand Brain Tissue

The first analysis was performed for all samples together to identify a common reliable reference gene. Then, we also analyzed brain and heart samples separately to see whether or not there was a difference between tissues.

Based on RefFinder, the 6 most reliable reference genes were the same for brain or heart samples. However, their ranks were a little different between heart and brain. For heart tissue, the order of best reference genes was as follows: U87 (GM = 1.97), U5a (GM = 2.45), 5S rRNA (GM = 3.03), U6 (GM = 3.36), GAPDH (GM = 3.81) and Z39 (GM-5.18) (Table 7). However, 5s rRNA (GM-1.73) was the top ranked gene when using samples from the brain tissue; the following best candidates were Z39 (GM = 2.00), U87 (GM = 2.66), U5a (GM = 2.78), U6 (GM 4.36), and GAPDH (GM = 6.48) (Table 8).

**Table 7:** A summary of ranking for reference gene candidates using 5 different statistical approaches.

RefFinder		dCt method		NormFinder		geNorm		BestKeeper			
								Ranking			
Genes	GM	Genes	SV	Gene name	SV	Gene name	SV		[r]		SD
**U87**	1.97	U87	1.3	GAPDH	0.64	5sRNA | U5a	0.18	HPRT	0.82	U87	0.21
**U5a**	2.45	U6	1.33	TNKS	0.66	Z39	0.24	UBC	0.73	U5a	0.32
**5sRNA**	3.03	U5a	1.34	U87	0.68	U6	0.26	TNKS	0.62	5sRNA	0.34
**U6**	3.36	5sRNA	1.35	U6	0.78	U87	0.35	U6	0.54	U6	0.37
**GAPDH**	3.81	GAPDH	1.39	UBC	0.79	GAPDH	0.59	GAPDH	0.49	Z39	0.46
**Z39**	5.18	Z39	1.43	U5a	0.82	U2	0.74	5sRNA	0.40	U2	0.70
**TNKS**	5.47	TNKS	1.44	5sRNA	0.85	TNKS	0.86	U5a	0.39	GAPDH	0.70
**UBC**	7.54	UBC	1.51	Z39	0.99	UBC	0.97	Z39	0.30	TNKS	0.90
**U2**	7.64	U2	1.54	U2	1.02	B2m	1.07	U87	0.27	UBC	0.97
**HPRT**	10.72	HPRT	1.72	HPRT	1.21	HPRT	1.19	U2	−0.22	HPRT	1.44
**B2m**	10.95	BACT	1.85	BACT	1.46	BACT	1.30				
**BACT**	11.72	B2m	1.86	B2m	1.50	18S	1.407				
**18S**	12.94	TBP	2.22	TBP	1.96	TBP	1.53				
**TBP**	13.49	18S	2.23	18S	2.01	GADD45A	1.68				
**GADD45A**	15	GADD45A	2.64	GADD45A	2.46						

24 samples were combined from heart experimental groups as input.

Geometric mean (GM); Stability Value (SV); Pearson’s correlation coefficient ([r]); Standard Deviation (SD).

**Table 8:** A summary of ranking for reference gene candidates using 5 different statistical approaches.

RefFinder		dCt		NormFinder		Genorm		BestKeeper				
								Ranking				
Genes	GM	Genes	SV	Gene name	SV	Gene name	SV			[r]		SD
**5sRNA**	1.73	5sRNA	1.34	Z39	0.82	5sRNA | U5a	0.11		5sRNA	0.82	U87	0.11
**Z39**	2.00	Z39	1.35	U87	0.83	U6	0.17		Z39	0.80	Z39	0.61
**U87**	2.66	U5a	1.37	5sRNA	0.84	Z39	0.21		U6	0.79	5sRNA	0.64
**U5a**	2.78	U6	1.4	UBC	0.86	U87	0.47		U5a	0.79	U5a	0.65
**U6**	4.36	U87	1.41	U5a	0.89	GAPDH	0.75		UBC	0.71	U6	0.70
**GAPDH**	6.48	UBC	1.53	U6	0.93	B2m	0.93		U2	−0.69	GAPDH	0.76
**UBC**	7.00	GAPDH	1.54	GAPDH	0.96	U2	1.04		HPRT	0.64	U2	0.87
**B2m**	8.37	HPRT	1.62	HPRT	1.04	TNKS	1.13		U87	−0.59	TNKS	0.98
**TNKS**	9.00	TNKS	1.65	TNKS	1.10	UBC	1.21		TNKS	0.28	UBC	1.02
**U2**	9.59	B2m	1.75	B2m	1.38	18S	1.29		GAPDH	0.16	HPRT	1.41
**HPRT**	9.80	U2	1.82	BACT	1.47	HPRT	1.36					
**18S**	11.72	18S	1.87	U2	1.48	BACT	1.46					
**BACT**	12.47	BACT	1.88	18S	1.54	GADD45A	1.55					
**GADD45A**	14.00	GADD45A	2.13	GADD45A	1.81	TBP	1.67					
**TBP**	15.00	TBP	2.45	TBP	2.20							

48 samples were combined from heart and brain experimental groups as input.

Geometric mean (GM); Stability Value (SV); Pearson’s correlation coefficient ([r]); Standard Deviation (SD).

Unlike the results from combined tissue, the first V-value<0.15 was at V2/3 (Figure 2). Thus, only 2 stable reference genes were needed for gene expression analysis in heart or brain tissue. A closer look at the data in Figure 2, the average of V-values for combined heart and brain tissue was 0.18. Individually, the average V-value for the heart and brain were 0.13 and 0.14, respectively. Thus, the gene candidates were merely more stably expressed in the heart tissue than in the brain tissues. However, though the genes’ expressions were consistent and stable within each tissue, it was different between the heart and brain. This can be inferred from the dramatic increase in the V-value average when both tissues were combined for analysis.

The top six most stable reference gene candidates were same for heart and brain tissues. The choice of the gene pair depends on the estimated expression levels of the targeted genes of interest. If the expression profiles of the genes of interest is unknown, then choosing reference candidates from the low and high extremes would be recommended such as U87 and 5S rRNA.

## Discussion

Housekeeping genes are commonly used for normalizing gene expression because they are thought to be consistently expressed cross different tissues and among different treatment. However, this was challenged recently. Current studies show no one gene remains stable throughout all experimental conditions. Ideal reference genes vary with different species, strains, developmental stage, tissue and even different sampling times [29]. To maintain the integrity of qRT-PCR as a powerful “discovery” and “validation” biotechnique, the choice and the number of reference genes used should be customized to every experiment setting. Thus, the first task is to identify reference gene candidates from either systematic gene expression studies like microarrays or by compiling a gene list from previous studies with similar experimental conditions. Subsequently, their relative stability is compared using statistical approaches. In our study, we followed the same workflow to determine reliable reference genes in SD rats. Normalizing to the top-ranked genes will reveal possible roles of hormonal/gender differences, mainly estrogen levels, in alcoholism. Below, we highlight the significance of our study by discussing some shortcomings associated with employing single statistical approaches in reference gene identification. In the end, we show the advantages of our combinatorial approach and present recommendations of control gene candidates to use or to avoid in similar experimental settings.

### Comparing RefFinder Results Across Tissue Combinations

The top 6 most stable reference candidates in the bi-tissue input were U87, 5S rRNA, GAPDH, U6, U5a, and UBC. In the single-tissue input, the top 6 were similar and only with a slight difference in the order. Results for the combined and separate inputs differed by only one reference gene ‘Z39’. This means that Z39 was stable within each tissue (SD<1), but its expression differed between heart (Ct_avg_ = 19.6±0.5) and brain (Ct_avg_ = 21.16±0.7). Moreover, U5a (ΔCt_avg_ = 1.64) and U6 (ΔCt_avg_ = 1.59) also had the highest Ct_avg_ difference between heart and brain tissue (Table 2). Even though the SD for Z39, U5a, and U6 was <1 in heart or brain tissue, their expression varied between heart and brain. Due to the inter-tissue variation, using Z39, U5a, and U6 in combined gene expression analysis is not recommended. In all input combinations, 18S rRNA, TBP, GADD45A, and BACT were the least stable among the 15 tested genes. These genes might cause inaccurate conclusions in gene expression normalizations for heart and/or brain tissue and therefore should not be used as reference genes.

### Comparing Among Different Methods and Tissue

**Figure 3:**
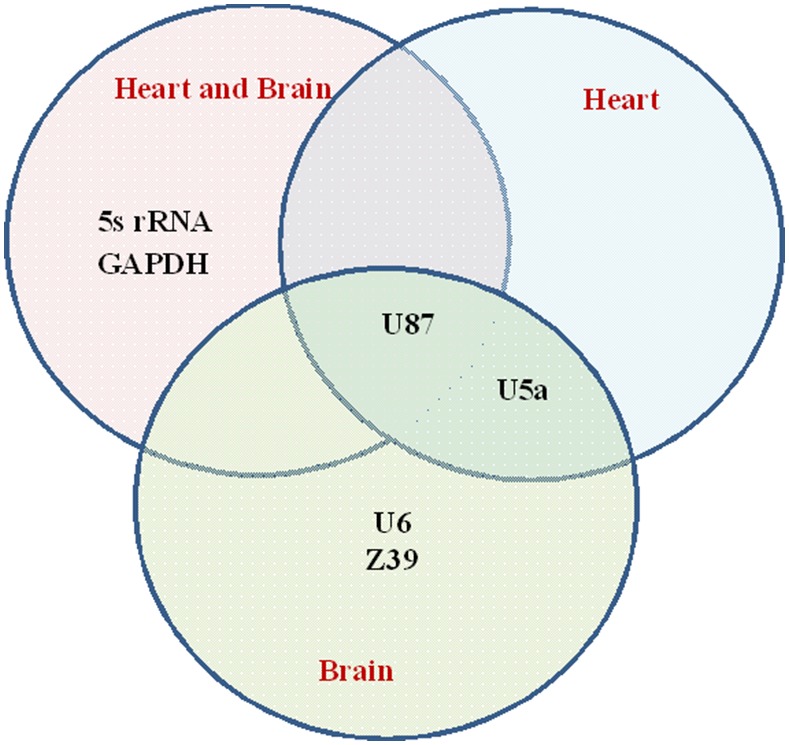
Van Diagram that summarizes the commonly ranked top gene candidates. Firstly, the top 6 genes ranked by each of the geNorm, dCt method, NormFinder, BestKeeper, and RefFinder were compared for each input: Heart+brain, heart, and brain samples. Only genes common for all 5 methods were chosen for each input. Those genes were then compared among all input combinations and presented in the diagram above.

When considering all brain and heart tissue samples together, U87, 5S rRNA, and GAPDH were commonly ranked among the top 6 most stable reference genes in all 5 statistical approaches. On the other hand, only ‘U87 and U5a’, and ‘U87, U5a, U6, and Z39’ were ubiquitously ranked among the top 6 across 5 methods in each of the heart tissue, and brain tissue, respectively. As shown in Figure 3, seniority of U87 was common to all statistical methods and all tissue combinations. If the targeted genes of interest are expressed at a lower level, we recommend the use of U87 with GAPDH for the two tissues. If the targeted genes are expressed at a higher level, then 5S rRNA and U87 would be better for normalization. U87 and U5a were commonly ranked among the best in each of heart and brain tissue. Thus, for gene expression analysis concerned with heart tissue or brain tissue, U87 and U5a would serve as better reference genes.

### Fallibility of Normalizations to Single Commonly Used HKGs

There is a wealth of resourceful studies that identified experiment-specific reference genes for normalization. We summarized some of the results for investigations that employed similar experimental conditions (i.e. rats, estrogen, and alcohol). Table 9 shows different HKGs as better reference genes for different tissue, treatments, treatment times, strains, species, and statistical methods. This suggests the necessity of conducting preliminary studies to use reference genes adapted for particular experimental conditions. In our study, U87, 5S rRNA, GAPDH and U5a were ranked as the top gene candidates using a combination of 5 statistical approaches. Even though, 5S rRNA was stable in rat liver treated with hepatotoxins [30], both U87 and 5S rRNA were among the least stable in SD rats suffering from oxygen-induced retinopathy [31]. While some studies reported GAPDH to be a relatively stable housekeeping gene in heart and brain tissue [29], [32]–[34], its expression was nevertheless affected by treatments such as MB in rat brain [35], estrogen in ovariectomized C57BL6 mice [36], male and female fathead minnow fish [37], and RARAW 264.7, ATDCDC5 and HFLS cells [38].

**Table 9:** A minireview of the reference gene candidates ranked as top or least stable in different experimental settings using higher organisms.

Modelsystem	Experimentalcondition	Statisticalmethod	GenesRanked		Reference
			Top	Least	
Young adult male Sprague-Dawley (SD) rats, New Zealand White (NZW) rabbits	Intervertebral disc (IVD)	geNorm, NormFinder,BestKeeper	HPRT1, CYCA		PMC 3118343 [45]
Fischer 344 (F344,resistant to OIR) and Sprague-Dawleyrats (SD, susceptible to OIR)(both albino inbred)	Oxygen-inducedretinopathy,Different strains, Differentdevelopment stage	Basic Statistics	U6,MIR-16	U87, 5S,4.5S	PMID 23441123, PMC 3580969 [31]
Timed-pregnantSprague-Dawley rats;	Dissected carotid body; Norm/hyper/hypo-oxial; Different developmental timing; Different strains	geNorm,NormFinderBestKeeper		18S, Actin	PMID 22023793[39]
Adult Sprague-Dawley rats	Surgically isolated 8 different liver cells at different times of liver regeneration	geNorm	ACTB, B2M, UBC	GAPDH	PMID 20339955[44]
TCDD-sensitive inbredLong-Evans rats	TCDD, Liver spleen hypothalamus	Basic Statistics	PGK1, GAPDH		PMID 16466705[32]
Sprague–Dawley (SD)neonatal + adult rat	Heart, PHDI treatment, Normoxia; hypoxia	geNorm,NormFinder	GAPDH,ACTB, B2M	TBP	PMC 3294216[33]
Wistar Rat brain cells: astrocytes andOLG cultures; OLG from mature +neonatal rats	No treatment, LXR agonist	geNorm;NormFinder	CYCA, PGK1,PRPL13A,YWHAZ,CYCA,PGK1,PRPL13A	GAPDH,18S, HMBS,GAPDH, 18S	PMID 20036692[40]
Obese Zucker rats	Heart	geNor	SDHA,TBP, HPRT1		PMID 22493144[43]
Obese Zucker rats	Kidney	geNor	TBP,GAPDH,ACTB		PMID 22493144[43]
Obese Zucker rats	Pulmonary	geNor	ACTB,YWHAG,SDHA		PMID 22493144[43]
Male flinders rats	Hippocampus, Methylene Blue	NormFinder;geNorm	YWHAZ, CYCA, RPL13A, HPRT1	GAPDH, ACTB, 18S	PMID 18241047 [35]
Male and femaleSprague-Dawley rats	Liver, Hypophysectomy	Basic Statistics		Tubulin,G3DPH,Bactin, TAT,Cyclophilin,18S	PMID 16724986[41]
Adult male Sprague-Dawley	Collagenase-intracerebral hemorrhage in RBG andLBG, 5 hr and 24 hrs	geNorm	GAPDH, HPRT, B2MG, GUSB		PMID 20089183[29]
Adult Male Wistar rats	Liver, Acetaminophen(AA),Carbon tetrachloride(CT),D-galactosamine (GA),Thioacetamide (TA)	geNorm;NormFinder;BestKeeper	MIR-16, 5S, B2M, 18S		PMID 22563491[30]
Adult male Sprague-Dawley rats	3 days following traumaticbrain injury (TBI),Cerebralcortex, Hippocampus	geNorm	HPRT, SDHA, GUSB, B2MG, TBP, GAPDH		PMID 18711751[34]
RARAW 264.7 (Mouse leukaemicmonocyte macrophage), ATDCDC5(chondrogenic) and HFLS (Human Fibroblast-Like Synoviocytes)	Estrogen			GAPDH	PMID 21472208[38]
Humans	Alcoholic liver disease	Basicstatistics	18S, SFRS4	Bactin, GAPDH	PMID 21913943[42]
C57BL6 mice	Ovariectomized, Uterus, Estradiol	BasicStatistics	RPL13A, 18S	GAPDH,HPRT1,PPIA,B2M,GUSB,ACTB,HSP90AB1	PMID: 19219570[36]
Male and female fathead minnow fish	Estrogen, Liver, Gonad	BasicStatistics	18S, RPL8, HPRT1,TBP	EF1A,G6PD,Bactin,GAPDH	PMID 17288598[37]

Basic Statistics: bi/multivariable; parametric/non-parametric hypothesis testing (e.g. t-test, ANOVA) and clustering methods.

On the contrary, 18S rRNA was among the least stable gene candidates in our settings. This is in agreement with other studies using carotid body from different SD strains under different oxygenation levels [39], oligodendrocyte cells from age-asynchronized Winstar rats treated with LXR agonist [40], male flinder rat hippocampus treated with MB [35], and liver of hypophysectomized male and female SD rats [41]. However, 18S rRNA was considered a good reference gene in Winstar rat livers treated with hepatotoxins [30], human livers with alcoholic liver disease [42], the uterus of ovarietomized C57BL6 mice treated with estradiol [36], and in liver and gonads of male and female fathead minnow fish [37]. We also showed that TBP was unstable and that was supported by another study on heart tissue of young and adult SD rats subjected to PHDI treatments under different oxygen pressures [33]. However, that was not the case in the heart of Zucker obese rats under different glycemic states [43], nor in the hippocampus of SD rats with TBI [34]. TBP was also considered stable in response to estrogen in multiple tissue of the fathead minnow fish [37].

### The Importance of Using More than One Statistical Approach

No one statistical approach can cover all variables associated with gene expression studies. Therefore basing conclusions on one method can be associated with false positive results and misleading conclusions. In our study, we followed a round-about approach to determine good reference candidates for reliable normalization of gene expression data in Sprague-Dawley rats. This allowed us to correct for some inaccurate ranking such as geNorm’s raking of UBC, which was corrected by NormFinder. Also, based on the systematic interpretation, we were able to get a clearer picture on the minimal number of reference genes required for reliable normalization. After removing Z39, U5a and U6, only 3 genes are enough to serve as reference genes for analysis on heart and brain tissue combined. This makes the study more practical (8 vs. 3 control genes) and reliable at the same time.

In conclusion, it is difficult to ascertain whether the inconsistency or variability in the stability of the housekeeping genes arises from the employment of different statistical methodologies or different treatments. For example, in two studies concerned with rat liver, GAPDH was stable after TCDD treatment based on geNorm [44], but it was not when using other statistical methods in liver cells under different conditions [32]. What is noteworthy is that the ranking of the reference genes is always relative and that can change simply by changing a few candidates in the gene list. Therefore, despite the superfluous publications, research concerned with the determination of reference remains juvenile. With more efforts being dedicated to tackle this issue, a meta-analysis would help reveal patterns that might redirect and standardize our normalization methods for more accurate interpretation of results.
